# Supplementary material for: Predicting willingness to be vaccinated for Covid-19: Evidence from New Zealand
Source: PLoS One. 2022 Apr 7;17(4):e0266485. doi: 10.1371/journal.pone.0266485 (PMC8989211; doi:10.1371/journal.pone.0266485)
Supplement: S1 Appendix — (PDF) [file pone.0266485.s003.pdf]

Appendix A – Sample demographics

Table A1. Age distribution of respondents

Age category (years)	Percentage of respondents	Percentage of New Zealand residents[1]
18–29	16.3	19.6
30–39	19.4	17.8
40–49	19.1	17.4
50–59	15.7	17.5
60–69	14.4	13.9
70 and over	14.8	13.7

Notes: [1]Source: [1]

Table A2. Distribution of respondents by highest educational qualification

Education category	Percentage of respondents	Percentage of New Zealand residents[1]
Some or all of secondary school	21.6	19.3
Certificate (1–6)	17.3	43.8
Diploma (5–7)	16.1	10.4
Graduate or postgraduate	45.0	26.4

Notes: [1]Source: [2]

Table A3. Ethnicity distribution of respondents

Ethnic category	Percentage of respondents	Percentage of New Zealand residents[1]
European	70.9	62.4
Māori	5.7	14.8
Pacific Islander	2.6	7.4
Asian	12.0	14.0
Other	7.6	1.4

Notes: [1]Source: [1]

Table A4. Income distribution of respondents

Income category	Percentage of respondents	Approximate percentage of New Zealand households[1]
Less than $20,000	6.4	10.0
$20,000 to $50,000	25.6	20.0
$50,000 to $70,000	15.8	20.0
More than $70,000	40.3	50.0

Notes: [1] Based on household income deciles. First decile <$25,400, second and third deciles $25,400 to $52,199, fourth and fifth deciles %52,200 to $82,999, remaining deciles >$83,000. Source: [3]

References

1. Stats NZ. Estimated resident population (ERP), subnational population by ethnic group, age, and sex, at 30 June 1996, 2001, 2006, 2013, and 2018 (2020). http://nzdotstat.stats.govt.nz/WBOS/Index.aspx?DataSetCode=TABLECODE7512#
2. Stats NZ. Highest qualification and ethnic group (grouped total responses) by age group and sex, for the census usually resident population count aged 15 years and over, 2006, 2013, and 2018 Censuses (2020). http://nzdotstat.stats.govt.nz/wbos/Index.aspx?_ga=2.69061078.636843804.1602117753761746062.1551927941#
3. Stats NZ. Household income and housing-cost statistics: Year ended June 2018 (2020). https://figure.nz/table/lWZNb5JQwzPrnOlD/download-source-dataset
