# Supplementary material for: Predicting willingness to be vaccinated for Covid-19: Evidence from New Zealand
Source: PLoS One. 2022 Apr 7;17(4):e0266485. doi: 10.1371/journal.pone.0266485 (PMC8989211; doi:10.1371/journal.pone.0266485)
Supplement: S2 Appendix — (PDF) [file pone.0266485.s004.pdf]

Appendix B: Belief segments results

Table B1. Belief segments for Covid-19

Statement	Covid-19 convinced (37%)	Covid-19 moderates (40%)	Covid-19 ambivalents (12%)	Covid-19 asymptomatics (5%)	Covid-19 sceptics (6%)
Coughing and sneezing spreads Covid-19	4.65	4.30 [a]	3.64 [a, b]	2.77 [a, b, c]	4.25 [a, c, d]
Covid-19 spreads from surfaces touched by infected people	4.35	3.84 [a]	3.25 [a, b]	2.06 [a, b, c]	4.11 [c, d]
Covid-19 is only a danger to the elderly and people with health problems	1.32	2.21 [a]	3.61 [a, b]	1.48 [b, c]	4.25 [a, b, c]
You are immune to re-infection once you have had Covid-19	1.95	2.47 [a]	2.86 [a, b]	1.77 [b, c]	4.07 [a, b, c, d]
Children cannot catch Covid-19	1.23	1.72 [a]	2.59 [a, b]	1.29 [b, c]	3.75 [a, b, c, d]
Children are perfectly safe from Covid-19	1.25	1.81 [a]	2.81 [a, b]	1.38 [b, c]	4.16 [a, b, c, d]
You cannot catch the virus from people without symptoms	1.27	2.09 [a]	2.90 [a, b]	1.40 [b, c]	3.98 [a, b, c, d]
Covid-19 is a hoax	1.11	1.53 [a]	2.61 [a, b]	1.21 [b, c]	3.97 [a, b, c, d]
Fears about Covid-19 are exaggerated	1.45	2.45 [a]	3.48 [a, b]	1.56 [b, c]	4.41 [a, b, c, d]
Covid-19 is no worse than the seasonal flu	1.33	2.23 [a]	3.09 [a, b]	1.48 [b, c]	4.07 [a, b, d]
Covid-19 is man-made	2.23	2.70 [a]	3.19 [a, b]	2.27 [b, c]	4.00[a, b, c, d]
Covid-19 comes from bats	2.96	2.79	2.67 [a]	2.40	4.10 [a, b, c, d]

Notes: Values are mean agreement ratings. Ratings ranged from a minimum of 1 (strongly disagree) to a maximum of 5 (strongly agree).

Differences in mean agreement ratings between segments tested using Tukey's HSD [1], ($p<0.01$)

[a] Mean differs from mean for the 'convinced' segment

[b] Mean differs from mean for the 'moderates' segment

[c] Mean differs from the mean for the 'ambivalents' segment

[d] Mean differs from the mean for the 'asymptomatics' segment

Table B2. Belief segments for eliminating Covid-19

Statement	Elimination enthusiasts (26%)	Elimination moderates (18%)	Vaccine hopefuls (34%)	Elimination sceptics (22%)
We need to eliminate Covid-19 to save lives	4.67	3.53 [a]	4.37 [a, b]	3.46 [a, c]
We should just live with it until we have a vaccine	1.44	2.72 [a]	2.63 [a,]	3.83 [a, b, c]
It would be better to let it spread and build herd immunity	1.26	2.19 [a]	1.69 [a, b]	3.70 [a, b, c]
There is no point trying to eliminate Covid-19 because it is a virus and will keep changing	1.66	2.49 [a]	2.76 [a, b]	3.99 [a, b, c]
Covid-19 is everywhere in the world so there is no way we can keep it out	1.71	2.26 [a]	3.80 [a, b]	4.16 [a, b, c]

Notes: Values are mean agreement ratings. Ratings ranged from a minimum of 1 (strongly disagree) to a maximum of 5 (strongly agree).
Differences in mean agreement ratings between segments tested using Tukey's HSD test [1] ($p<0.01$)
[a] Mean differs from mean for the 'enthusiasts' segment
[b] Mean differs from mean for the 'moderates' segment
[c] Mean differs from the mean for the 'hopefuls' segment

Table B3. Belief segments for Covid-19 vaccination

Statement	Vaccination enthusiasts (24%)	Vaccination moderates (33%)	Vaccination ambivalent (28%)	Vaccination cautious (7%)	Vaccination sceptics (8%)
A vaccine will give lifelong protection against Covid-19	2.46	2.76 [a]	2.76 [a]	4.04 [a, b, c]	1.46 [a, b, c, d]
Getting vaccinated against Covid-19 means you will recover faster	3.58	3.39 [a]	2.98 [a, b]	4.08 [a, b, c]	2.14 [a, b, c, d]
Getting vaccinated against Covid-19 means your symptoms will be much weaker if you do get the virus	3.76	3.48 [a]	3.25 [a, b]	4.11 [a, b, c]	2.63 [a, b, c, d]
Once you are vaccinated you cannot catch Covid-19	2.51	2.74 [a]	2.63	4.19 [a, b, c]	1.60 [a, b, c, d]
Once you are vaccinated you cannot spread Covid-19	2.75	3.02 [a]	2.87	3.95 [a, b, c]	1.68 [a, b, c, d]
You should only have to get vaccinated against Covid-19 if you are old or have a health problem	1.16	2.02 [a]	2.76 [a, b]	4.05 [a, b, c]	2.79 [a, b, d]
Children shouldn't be vaccinated against Covid-19	1.70	2.25 [a]	2.94 [a, b]	3.87 [a, b, c]	3.71 [a, b, c]
Getting vaccinated against Covid-19 is a waste of time and effort	1.07	1.69 [a]	2.62 [a, b]	3.89 [a, b, c]	3.46 [a, b, c, d]
People who want to be vaccinated against Covid-19 are over-reacting	1.17	2.09 [a]	2.66 [a, b]	4.11 [a, b, c]	3.09[a, b, c, d]

Notes: Values are mean agreement ratings. Ratings ranged from a minimum of 1 (strongly disagree) to a maximum of 5 (strongly agree). Differences in mean agreement ratings between segments tested using Tukey's HSD [1], ($p<0.01$)

[a] Mean differs from mean for the 'enthusiasts' segment

[b] Mean differs from mean for the 'moderates' segment

[c] Mean differs from the mean for the 'ambivalents' segment

[d] Mean differs from the mean for the 'cautious' segment

Table B3. Belief segments for Covid-19 vaccination (continued)

Statement	Vaccination enthusiasts (24%)	Vaccination moderates (33%)	Vaccination ambivalent (28%)	Vaccination cautious (7%)	Vaccination sceptics (8%)
Getting vaccinated against Covid-19 should be compulsory	3.97	3.57 [a]	2.54 [a, b]	4.05 [b, c]	2.79 [a, b, d]
Vaccination against Covid-19 should be free	4.81	4.44 [a]	4.04 [a, b]	4.23 [a]	4.10[a, b]
It isn't worth getting vaccinated against Covid-19 yet as there are too many unknowns about the vaccines	1.43	2.35 [a]	3.41 [a, b]	4.27 [a, b, c]	4.53 [a, b, c]
I think we should wait and see if the Covid-19 vaccination works overseas before trying it here	1.75	2.59 [a]	3.70 [a, b]	3.93 [a, b]	4.19 [a, b, c]
Getting vaccinated against Covid-19 is unsafe because of the potential side effects	1.46	2.29 [a]	3.22 [a, b]	3.96 [a, b, c]	4.10 [a, b, c]
Getting vaccinated against Covid-19 is just not practical	1.15	1.95 [a]	2.71 [a, b]	4.13 [a, b, c]	3.26 [a, b, c, d]
Getting vaccinated against Covid-19 isn't worthwhile if you are only protected for a few months	1.70	2.52 [a]	3.44 [a, b]	4.07 [a, b, c]	4.09 [a, b, c]

Notes: Values are mean agreement ratings. Ratings ranged from a minimum of 1 (strongly disagree) to a maximum of 5 (strongly agree).
Differences in mean agreement ratings between segments tested using Tukey's HSD [1], ($p<0.01$)
[a] Mean differs from mean for the 'enthusiasts' segment
[b] Mean differs from mean for the 'moderates' segment
[c] Mean differs from the mean for the 'ambivalents' segment
[d] Mean differs from the mean for the 'cautious' segmen

Table B4. Vaccination belief segments and attitude towards being vaccinated

Segment	Right thing to do	Doesn't matter to me	Not sure	Haven't given it much thought	Bad thing to do
Vaccination enthusiasts	99.6	0.0	0.0	0.4	0.0
Vaccination moderates	90.9	4.0	4.8	1.2	0.0
Vaccination cautious	54.7	21.3	12.0	5.3	6.7
Vaccination ambivalent	33.6	9.6	47.1	7.5	2.1
Vaccination sceptics	0.0	6.3	48.8	7.5	37.5

Notes: Values are percentages of respondents in each segment. Test for differences in percentages across segments ($\chi^2 = 726.2$, $p < 0.01$)

Table B5. Vaccination belief segments and willingness to be vaccinated

Segment	Definitely	Probably	Maybe	Probably not	Definitely not
Vaccination enthusiasts	92.4	7.2	0.4	0.0	0.0
Vaccination moderates	66.7	27.0	5.2	1.2	0.0
Vaccination cautious	44.0	32.0	13.3	9.3	1.0
Vaccination ambivalent	13.6	21.8	46.4	13.2	5.0
Vaccination sceptics	0.0	0.0	18.8	26.3	55.0

Notes: Values are percentages of respondents in each segment. Test for differences in percentages across segments ($\chi^2 = 427.8$, $p < 0.01$).

Table B6. Vaccination belief segments and willingness to be vaccinated as soon as possible

Segment	Yes	Not sure	No
Vaccination enthusiasts	91.1	5.5	3.4
Vaccination moderates	76.7	18.1	5.2
Vaccination cautious	82.1	6.0	11.9
Vaccination ambivalent	23.1	49.3	27.5
Vaccination sceptics	0.0	26.7	73.3

Notes: Values are percentages of respondents in each segment who indicated they definitely, probably or maybe would get vaccinated for Covid-19.

Test for differences in percentages across segments (χ^2 = 339.8, p < 0.01).

Table B7. Vaccination belief segments and willingness to be vaccinated if it offers only temporary protection

Segment	Yes	Not sure	No
Vaccination enthusiasts	82.5	13.5	3.9
Vaccination moderates	60.5	28.2	11.3
Vaccination cautious	86.4	5.1	8.5
Vaccination ambivalent	19.3	48.8	31.9
Vaccination sceptics	0.0	50.0	50.0

Notes: Values are percentages of respondents in each segment who indicated they would get vaccinated as soon as possible for Covid-19.

Test for differences in percentages across segments ($\chi 2$ = 194.7, p < 0.01).

Table B8. Vaccination belief segments and bad experience with vaccinations

Segment	Yes	Not sure	No
Vaccination enthusiasts	5.1	2.1	92.8
Vaccination moderates	7.9	2.1	90.0
Vaccination cautious	42.7	8.2	56.0
Vaccination ambivalent	13.6	3.9	78.2
Vaccination sceptics	32.5	1.3	66.3

Notes: Values are percentages of respondents in each segment.

Test for differences in percentages across segments (χ2 = 126.2, p < 0.01).

Table B9. Vaccination belief segments and knowing someone who had a bad experience with vaccinations

Segment	Yes	Not sure	No
Vaccination enthusiasts	15.6	4.2	80.2
Vaccination moderates	17.9	3.6	78.5
Vaccination cautious	46.7	2.7	50.7
Vaccination ambivalent	25.0	11.8	63.2
Vaccination sceptics	24.6	6.3	69.2

Notes: Values are percentages of respondents in each segment.

Test for differences in percentages across segments (χ^2 = 107.9, p < 0.01)

Reference

1. Tukey J. Comparing individual means in the analysis of variance. Biometrics. 1949; 5(2): 99-114. doi:10.2307/3001913
