# Supplementary material for: Predicting willingness to be vaccinated for Covid-19: Evidence from New Zealand
Source: PLoS One. 2022 Apr 7;17(4):e0266485. doi: 10.1371/journal.pone.0266485 (PMC8989211; doi:10.1371/journal.pone.0266485)
Supplement: Attachment — Submitted filename: Response to reviewers january.docx [file pone.0266485.s005.docx]

Dear Editor,

Thank you for providing us with the opportunity to revise and resubmit our manuscript. I have entered a revised manuscript with and without ‘track’ changes as requested.

You raised the following you wished us to address:

- Specifically, in the revised version of the paper please better describe the framework used and the conducted analysis. The writing flow in some key parts of the paper - quality of the analysis and concluding remarks - is very difficult to follow. When submitting the revised version of the paper, please consider the reviewers' comments.

We have revised the manuscript in accord with reviewers’ comments, focussing in particular on the methods and results sections. Our revisions to the reviewers’ comments are described in detail below.

Please do not hesitate to contact us if there is anything else you require.

Kind regards

Dr Geoff Kaine.

Reviewer #2 comments:

**1. I found the discussion of the I3 framework a little difficult to follow until I got to Figure 1 and the examples in the context of vaccine hesitancy. Perhaps the authors could provide the example of vaccine hesitancy together with their explanation rather than after it. Alternatively, they can provide a briefer general explanation and then give examples sooner.**

*We had provided examples with respect to vaccine hesitancy in the Theory section where we introduce the I3 model. We have made the link between vaccine hesitancy and the I3 model clearer by referring specifically to the 5C model in that section. See revised lines 95, 100, 191, 213 and 268 (in the manuscript with track changes).*

**2. The authors transition from discussing their framework to COVID-19 in New Zealand to the methods section. Since the paragraph explaining the study itself is on page 4, nine pages earlier, the reader may forget the purpose of the study. I suggest adding a short paragraph explaining what was done and why before turning to the methods section.**

*We have added a short paragraph at the commencement of the Methods sections describing the purpose of the study as suggested (lines 308-312* *in the manuscript with track changes). We have also added a description of the steps in the analysis, and the purpose of each step, to the Methods section (lines 432-463 in the manuscript with track changes).*

**3. The paragraph on instrumentation was a little confusing. The authors say that there was a scale measuring involvement, but then that the order of the involvement, attitude, and belief scales was randomized. How were attitudes and belief measured? Then, the authors discuss how the attitudes scores were calculated but I could not find the scales used. It would be easier to follow if the authors had a separate paragraph for each measure and the traditional sections for instrumentation, sample, procedure, etc., though the methods section could be made clearer without using this format.**

*We have added additional material to the Methods section to provide more clarity on measurement methods (lines 320-324, 329-349 in the manuscript with track changes).*

**4. The authors provided information on the status of COVID-19 measures in New Zealand at the time of data collection, which is important. What was the vaccination status at the time? Were vaccines available at the time?**

*We have revised the relevant paragraph* *(lines 300-305 in manuscript with track changes) to be clear the study was conducted before vaccines were made available to the general public in New Zealand.*

**5. The authors wrote several paragraphs about self-report bias in the methods section. While this presents an interesting question, usually it belongs in the discussion section. Moving these paragraphs to the discussion will again make the manuscript clearer.**

*We have shifted the relevant paragraphs to the Discussion section (lines 998-1036 in the manuscript with track changes).*

**6. I did not understand how the respondents were classified into belief segments. Ward’s method implies a cluster analysis was used, is that so? Was this method used in other contexts using this scale, and were any validation measures used? This seems to be the main finding of the study and the results describe the clustering, so it should be presented in more detail.**

*We have revised the Methods section (lines 441-450 in the manuscript with track changes) and the reporting of the belief segments results to make it clear that cluster analysis was employed (lines 583, 594, 609, 621 in the* *manuscript with track changes). We have also added material to the Methods section to explain the steps in the analysis (lines 432-463 in the manuscript with track changes) and to clarify the purpose of the belief segment analysis was simply to condense the belief data into a smaller set of dummy variables for use in the regression analyses. This clarifies that the main finding of the study concern the regression results (and not the belief segment results).*

**7. I suggest** **adding standard deviations to the tables when relevant.**

*In our opinion adding standard deviations to the tables is unnecessary as we already report significance test results in all the relevant tables (which are themselves based on standard deviations), thereby saving the reader the effort of interpreting the results themselves.*

**8. The belief segments section explains why the study was conducted and the analytic approach. I think these should be in the introduction and methods section, respectively, as exploring the belief segments is a part of the study, again adding the aforementioned details about the cluster analysis. Alternatively, the authors may split the results into “study 1” and “study 2” if they feel like the studies are separate. There are similar problems in other parts of the results section.**

*We have added material to the Methods section to explain the steps in the analysis (lines 432-463 in the manuscript with track changes) and to clarify the purpose of the belief segment analysis was to condense the belief data into a smaller set of dummy variables for use in the regression analyses. Hence splitting the results into separate studies was not appropriate. We have revised the opening paragraphs of the belief segments section accordingly (lines 574-581 in the manuscript with track changes).*

**9. I suggest reporting the standardized coefficients in the regression tables to facilitate interpretability.**

*We have revised the relevant tables to report standardised regression coefficients for OLS regressions (Tables 9-13).*

**Minor comments**

**1. On page 4, I find it odd that the authors discuss the results before presenting them. I think the authors should discuss the framework and its importance, not specific results.**

*We have removed the two paragraphs describing the results from the Introduction* *(lines 73-91 in the manuscript with track changes).*

**2. Figure 1 should be of higher quality.**

*We have revised the Figure.*

Reviewer #3 comments:

**Please put more specific information on the use of each statistical test/method used in the analysis in the Materials and Methods section, such as the use of Cronbach's Alpha (page 19, line 429), Chi-Square test (pages 19-20, 22, lines 436, 441, 448, 457, 496-498), Tukey's HSD test (pages 22, 23, lines 488, 495), Binary Logistic Regression (page 38, lines 746, 747, 748), Nagelkerke R Square (page 38, line 753), to give some knowledge to the audience/readers so that your research may be more reproducible.**

*We have indicated where we have used these tests and methods in the additional material in the Methods section (lines 432-463 in the manuscript with track changes).*
