# Supplementary material for: Predicting willingness to be vaccinated for Covid-19: Evidence from New Zealand
Source: PLoS One. 2022 Apr 7;17(4):e0266485. doi: 10.1371/journal.pone.0266485 (PMC8989211; doi:10.1371/journal.pone.0266485)
Supplement: Attachment — Submitted filename: Response to reviewers march.docx [file pone.0266485.s006.docx]

Dear Editor,

Thank you for providing us with the opportunity to revise and resubmit our manuscript. I have entered a revised manuscript with and without ‘track’ changes as requested.

You raised the following point in your letter:

- Please note that reviewer 1 has requested citations which have limited relevance to the study topic. As such we suggest that you carefully review whether the suggested references further contribute to the literature discussion of your study. Please feel free to ignore any referenced without direct relevance.

We believe the references suggested by Reviewer 1 are not at all relevant to our study and so have not included them in the manuscript.

We have revised the manuscript in accord with the comments from Reviewer 2. We really appreciate the effort Reviewer 2 has put into their detailed comments. Our revisions in response to their comments are described below.

Please do not hesitate to contact us if there is anything else you require.

Kind regards

Dr Geoff Kaine.

Reviewer #2 comments:

**1. The paper still suffers from minor organization issues. For example, the authors introduce the 5C framework but then say that the study uses the I3 framework, without explaining what it is and how they are connected until much later, or discuss the 5C model** **among people in quadrant 3 with unfavorable attitudes towards the measure before discussing those attitudes.**

*We have replaced the discussion of the 5C model in the Introduction with a short statement referring to vaccine hesitancy generally. We introduce the components of vaccine hesitancy in the description of the I_3_ Framework (quadrant 1 in particular) to assist the reader to better understand the implications of Framework. See revised lines 46-61, 124-125 and 143-159* *(in the manuscript with track changes). We hope the restructure improves the readability of the manuscript.*

*Regarding attitudes towards being vaccinated among people in quadrant 3. The discussion here is intended to illustrative and we are suggesting some hypotheses about these people regarding the components of vaccine hesitance, simply on the basis of their involvement and whether their attitude is favourable or unfavourable. Hopefully we have made the illustrative nature of this discussion clearer by amending lines 214-215 (in the manuscript with track changes).*

**2. The way I understand Kaine et al. (2010), involvement with the issue in this context does not necessarily mean wanting to prevent the spread of COVID, but rather interest and research on the topic and involvement in decision-making related to it. One might conduct their own research and deduce that COVID is not very dangerous, an option addressed in the scales used in this study. This places them in the third quadrant (with unfavorable views), however, the description of this quadrant does not fit them. Could the authors please clarify the meaning of involvement with the issue, and if I understood correctly, adjust the quadrants’ descriptions accordingly?**

*We believe the reviewer is confusing involvement with Covid-19 per se and involvement with the policy outcome of eliminating Covid-19 from New Zealand. We have revised the definition of involvement with the policy outcome to clarify we are considering involvement with the outcome of elimination (lines 106-109 in the manuscript with track changes). Consequently, the quadrant descriptions do not require changing.*

**3. On page 7 line 158: “because people in this quadrant are uninterested, they are unlikely to pay attention to promotional and educational messaging, so the final strategy of promoting awareness is likely to be problematic.” I do not understand why promoting awareness is likely to be problematic. Do the authors mean ineffective? Or that promoting awareness is likely to cause resistance to the measure? This can be made clearer.**

*We have changed ‘problematic’ to ‘ineffective’ (line 177 in the manuscript with track changes).*

**4. On page 8 line 171: it seems to me that people with high involvement with the policy outcome will not be complacent with respect to the risk. They are familiar for example with COVID and its risks and are concerned about it, but are not familiar with the vaccine as the intervention. Perhaps the authors should explain why they think people in the different quadrants have these levels of the C5**

*We have revised the relevant paragraph* *(lines 191-192 in manuscript with track changes) to include reasons why people with high involvement in the policy outcome may be complacent with respect to risk of infection.*

**5. On page 9 line 188: “Consequently, a strategy for promoting compliance among individuals in this quadrant with a favourable attitude might focus on self-regulation, using mechanisms such as voluntary codes of conduct.” I am not sure I understand what voluntary codes of conduct mean in this case, and why they are necessary if the people in question are already likely to comply.**

*Codes of conduct may be an appropriate mechanism in other contexts (e.g. occupational health and safety) but are not relevant in this context as the reviewer points out. We thought the simplest solution was to delete mention of this mechanism from the paragraph (line 209 in the manuscript with track changes).*

**6.** **In quadrants 3 and 4, the authors assume that collective responsibility involves getting vaccinated. This may not be the case; some of those opposing the vaccine believe it is better for people to not get vaccinated (e.g., they believe the vaccines are dangerous to young people, again an option mentioned in the current survey). If the authors argue that collective responsibility is only linked to adopting the measure rather than other altruistic intentions (even if misguided), they should clarify this point.**

*In models of vaccine hesitancy such as the 5C model, ‘collective responsibility’ refers specifically to ‘a* *willingness to protect others by getting vaccinated’* *(see line 155 in the manuscript with track changes). We have added this qualifier at lines 221 and 282 in the manuscript with track changes).*

**7. I believe that the discussion of enforcing vaccines should be done more carefully given the current debate on the issue. The other alternatives for increasing compliance among Q3 people with unfavorable attitudes towards COVID vaccines are dismissed as ineffective, but not enforcement. This topic is well-covered in the discussion, so I think the introduction should also present the compliance issues with enforcement.**

*In the theory section we describe a number of alternatives for increasing compliance among Q3 with unfavourable attitudes with enforcement mentioned as one alternative. We did not conclude the alternatives to enforcement would be ineffective., though we did note they might be unsuccessful if people engage in motivated reasoning. To avoid appearing to advocate enforcement too forcefully we have qualified the suggestion of enforcement in relation to Q3 (and Q4) with unfavourable attitudes by noting the imposition of blanket penalties for non-compliance may run the risk of incidentally alienating people with low involvement (see lines 242-244 and 269-271 in the manuscript with track changes).*

*We did note that if the causes of non-compliance relate to unpredictable variations in the environment, or to unforeseeable technical problems, then enforcement and general deterrence may be ineffective, and the provision of technical assistance would be more appropriate; one could imagine this might translate into alternative healthcare arrangements for example (see lines 246-249 in the manuscript with track changes).*

**8. It is unclear to me how someone can have favorable attitudes towards the COVID-19 vaccine without being involved with wanting to prevent the disease. Perhaps the authors could provide an example?**

*We presume the Reviewer is referring to the possibility of a person in quadrant 4 with a favourable attitude towards vaccination but low involvement with eliminating Covid-19. The key here is that having a favourable attitude does not necessarily mean one is highly motivated. One may have a favourable attitude towards vaccination, that does not mean one necessarily cares strongly about ‘eliminating Covid-19 from New Zealand’. For example, a person who views vaccination purely in terms of personal protection may favour vaccination but be disinterested in the outcome of eliminating Covid-19. Presumably such a person will have a limited sense of collective responsibility in the 5C sense. We have amended the relevant paragraph to include this example (see lines 277-278 in the manuscript with track changes) and thank the reviewer for bringing this opportunity to clarify to our attention.*

**9. I thank the authors for providing the information about vaccinations in New Zealand.**

**10.** **The authors introduce the five components of involvement in the methods section, but this was not mentioned before. If the authors are not interested in presenting these components in detail in the introduction, they can briefly describe each component in the methods.**

*We have added a brief definition of the five components of involvement to the Methods section (see lines 327-332 in the manuscript with track changes).*

**11. Based on the description of the measures, it seems like there was only one measure of involvement. The supplement material has two involvement scales, which makes it clear how the quadrants were determined. In order to help the reader who may not read the supplement material, I suggest clarifying that both types of involvement were measured.**

*We have revised the relevant paragraph to be clear both types of involvement were measured (see lines 323-324 in the manuscript with track changes).*

**12.** **In the first stage of analysis, there seems to be no separation between favorable and unfavorable attitudes towards the vaccine in the relevant quadrants. Was this planned or due to the small proportion of unfavorable attitudes in the sample?**

*The proportion of respondents with favourable and unfavourable attitudes towards vaccination in each quadrant is reported in Table 3. As the reviewer observes, fundamentally, the proportion of unfavourable responses in the sample was too small to generate significant results. Furthermore, the data used to generate the results reported in Tables 5 and 6 was restricted to respondents who indicated that they definitely would, probably would, or might get vaccinated.*

**13.** **I suggest that the authors use item means in their tables instead of the scores on the individual items. This will help reduce the number of tables and make them clearer.**

*We believe the reviewer is referring to Tables 8 and 9 where we present results showing differences across the quadrants with respect to items from the survey that we believe related to the components of the 5C model. We prefer not to summarise these tables as suggested because the variables were not intended to be scales for measuring the 5C components and are not the standard scale items used in 5C model (these qualifications are made at lines 520-522 in the manuscript with track changes). By presenting the mean scores for each variable (rather than aggregating them into scales) the reader is in a position to judge for themselves whether the variables we report reflect the 5C components and judge the results accordingly.*

**14. On page 28: since the authors explain the clustering technique in the methods section, they do not have to mention it again in the results section.**

*We have revised the relevant paragraphs in the Methods and Results to remove the repetition (see lines 427-429 and lines 563-566 in the manuscript with track changes).*

**15. I thank the authors for including the standardized coefficients in their tables reporting the regression results. Note that when reporting standardized coefficients, the intercept is always zero. If the authors want to include the intercept, they should have separate columns for standardized and unstandardized parameters.**

*We have removed reporting of intercepts in the relevant tables (Tables 9-13 in the manuscript with track changes).*

**16. If the benchmark group is skeptics, then the results’ description should reflect that. For example, respondents with ambivalent beliefs about COVID had less favorable attitudes in comparison to the skeptics, not all of the other respondents.**

*We have revised the relevant paragraph Results to state beliefs about Covid-19 itself did not strongly influence attitude toward eliminating Covid-19 in New Zealand over and above beliefs about elimination (see lines 736-739 in the manuscript with track changes).*
